# TCR Signaling Abnormalities in Human Th2-Associated Atopic Disease

**DOI:** 10.3389/fimmu.2018.00719

**Published:** 2018-04-16

**Authors:** Joshua D. Milner

**Affiliations:** Laboratory of Allergic Diseases, National Institute of Allergy and Infectious Diseases (NIAID), National Institutes of Health (NIH), Bethesda, MD, United States

**Keywords:** T-cell receptor repertoire, signaling pathways, primary immunodeficiencies, atopic disease, monogenic syndromes

## Abstract

Stimulation of naïve CD4 T cells with weak T cell receptor agonists even in the absence of T helper-skewing cytokines can result in IL-4 production which can drive a Th2 response. Evidence for the *in vivo* consequences of such a phenomenon can be found in a number of mouse models and, importantly, a series of monogenic human diseases associated with significant atopy which are caused by mutations in the T cell receptor signaling cascade. Such diseases can help understand how Th2 responses evolve in humans, and potentially provide insight into therapeutic interventions.

## Introduction

Within the vast legacy of Bill Paul’s career, one theme that emerged was the search for a source of IL-4 that would meaningfully provide differentiating naïve CD4 T cells sufficient signal to develop into memory Th2 cells during an immune response that required such a program. Mast cell (1), basophil (2–4), and NK T (5) IL-4 production were observed, but whether they are the key initiators of most Th2 responses continues to be a matter of debate. It was therefore in the course of that search that attention was turned to IL-4 production by the naïve T cell itself (6, 7). Kim Bottomly, herself a trainee of Bill’s, had observed that *in vitro* priming of naïve T cells by relatively weak, but not strong, agonist peptides, in the absence of other priming cytokines, could lead to a Th2 response (8–15). Bill’s lab later showed that “strong” agonist peptides themselves could prime such a response, when provided at a sufficiently low dose (16), and that in lymphopenic states or when TCR of high affinity for a given peptide are removed, stimulated naïve cells will differentiate into Th2 cells (17, 18). The mechanisms for these observations continue to be unraveled, but include the notion that responses to IL-2 become blunted at higher dose of peptide, preventing the necessary STAT5b activation and nuclear translocation for transcription of key Th2 lineage transcription factor as well as poor ERK activation, as MEK inhibition could recapitulate the Th2 bias even in the presence of high dose strong TCR agonism (9, 16).

*In vivo*, indeed TCR/MHC interactions may even predominate over exogenous adjuvant activity in determining Th1/Th2 balance (19), although it may not always be *via* IL-4 production itself (20). One potential teleologic reason for the phenomenon could be that parasitic products which could evade immune responses by downregulating TCR-MHC interactions [such as the omega-1 component of schistosome egg antigen which can prime Th2 responses, potentially by weakening TCR/MHC interactions (21, 22)] resulted in the evolution of anti-parasitic cytokine profiles which are derived from differentiation under low-affinity conditions. Whatever the cause, and whether IL-4 itself is the key driver of Th2 differentiation *in vivo* is a matter of debate, the success of IL-4 receptor blocking antibodies in treating human atopic disease has been impressive, strongly suggesting this pathway is critical for the pathogenesis of human atopic disease (23).

Another set of observations have further buttressed the notion that altered TCR signaling could lead to Th2 phenotypes. A series of mouse lines derived spontaneously or *via* random mutagenesis with missense mutations in key TCR signaling molecules were observed to develop Th2-related pathology spontaneously. These included LAT, ZAP70 (in several independent mutant lines), and CARMA1 (24–28). Null mutations in most of these molecules lead to impairment of effector function which precludes most Th differentiation altogether, and as such it is the hyopomorphic loss-of-function mutations which lead to the phenotype.

Of course, a major consequence of this basic observation could be that certain human disease could also be driven by this phenomenon and would most likely include an atopic phenotype. With the exponential growth of patients undergoing next-generation sequencing, multiple newly described immune disorders which include atopic disease have been identified, some of which may well be due to impaired TCR signaling. This review therefore provides a series of examples of human monogenic disorders associated with atopy which may be caused by imbalances in TCR signaling which fail to prevent Th2 responses.

## Omenn Syndrome (OS)

Before directly addressing the propensity for mutations to intrinsically bias a T cell toward Th2 differentiation, it is critical to distinguish one congenital atopic phenotype, namely, that seen in OS (29). Mutations that are known to lead to massive curtailment of T cell function and/or number—both intrinsic to signaling and extrinsic to it—can nonetheless permit “leaky” peripheral T cell populations which can progress to CD4 lymphoproliferation, organomegaly, and Th2-like disease associated with marked IgE elevation, erythroderma, and eosinophilia. Why OS is associated with the Th2 phenotype is not clear, but hypotheses have included a failure of central tolerance due to abnormal thymic development which hinders both AIRE-induced negative selection and the generation of a normal repertoire of FOXP3+ regulatory T cells (Tregs) (30–32). The lymphopenic state also may lead to the absence of sufficient high-affinity competition for antigen which would then permit low-affinity cells to be stimulated and proliferate, leading to the Th2 phenotype (17, 18).

## Mutations in Genes Encoding Classical TCR Signaling Proteins

Similar to the mouse, human mutations in ZAP70 can lead to varied phenotypes from SCID, to autoimmunity, to highly atopic phenotypes (33–37). In the case of one of the reported atopic phenotype in humans, it is not clear whether it was caused by intrinsic Th2 bias similar to the mouse model, or due to the limited repertoire associated with OS (35).

Stronger evidence for the link between TCR intrinsic signaling defects and atopy in human disease can be found in hypomorphic mutations of two members of the CBM complex, such as MALT1 (38) and CARMA1 (39, 40). The CBM complex, which includes MALT1, CARD11, and BCL10, is required for normal NFkB activation after TCR ligation, as well as mTORC1 activation (41, 42). Complete loss-of-function mutations of any of the three CBM complex members lead to a SCID-like illness (43–47), but recently, hypomorphic MALT1 mutations were described in a patient with recurrent infection, marked IgE elevation, and severe eczema (38). Even more recently, dominant-negative mutations leaving residual, hypomorphic CARD11 activity were identified in a cohort of patients with severe atopic disease with, and in some cases, without, comorbid infection. The finding is of particular interest since, in addition to the possibility that severe atopy without comorbidity could be explained by a single-gene mutation, CARD11 has been identified in GWAS studies of common atopic dermatitis (48).

While numerous patients with defects in nearly every NFkB subunit have been identified, atopy has not been reported to be associated with any of them. The lack of atopy argues that defects in another pathway in which CARD11 is involved might explain the allergic disease these patients have. Recent evidence suggests that CARD11 may also participate in mTORC1 activation (42) by recruiting, upregulating, and/or activating of the glutamine transporter ASCT2, which in turn leads to increased intracellular glutamine needed for mTORC1 activation. ASCT2−/− mice have a Th2 phenotype (49), potentially due to inadequate glutamine transport, which may be required for normal Th1 differentiation and the prevention of excessive Th2 differentiation (50, 51). The CARD11DN patients have evidence of impaired mTORC1 activation and reduced Th1 cytokine production, rescuable by exogenous glutamine (39), raising the possibility that glutamine supplementation could be of clinical benefit in these patients. Of note, glutamine supplementation of premature infants is associated with protection from the development of atopic dermatitis (52, 53).

## Mutations in Genes Encoding Actin Cytoskeleton Proteins

Following TCR ligation, Wiskott–Aldrich syndrome protein (WASP) dissociates from its stabilizing partner WASP-interacting protein (WIP) and binds actin-related protein (ARP) 2/3 (54) to begin the actin assembly cascade.

Loss of WASP leads to Wiskott–Aldrich syndrome, which is characterized by severe atopic dermatitis, increased gut sensitization and clinical food allergy, thrombocytopenia, and combined immunodeficiency (55, 56). A similar phenotype occurs with loss of WASP-interacting protein family member 1 (*WIPF1*) encoding WIP (57) as well as an ARP2/3 subunit, actin-related protein 2/3 complex subunit 1B (*ARPC1B*) (58–60).

WASP-interacting protein also appears to associate with dedicator of cytokinesis 8 (DOCK8) a guanine nucleotide exchange factor whose activity is critical for normal WASP function (61). Loss of function in DOCK8 leads to significant elevations in IgE, combined immunodeficiency, and other many clinical features in common with WAS, including severe atopic dermatitis and food allergy, and even autoimmunity (62, 63). Thrombocytopenia is not seen in DOCK8 deficiency, while severe viral skin infections and anaphylaxis are not as common in WAS, potentially due to differences in redundancy, function, and tissue expression (56, 64, 65).

Once again, we know less about why Th2 phenotypes emerge from these actin cytoskeleton-related mutations. DOCK8 patient lymphocytes have a T cell-intrinsic bias toward Th2, and away from Th1 differentiation (66), and WASP transcriptional activity appears to be critical for Th1 differentiation (67, 68). Another possible mechanism suggests these proteins have critical roles in Treg function, potentially *via* IL-2 activity, the impairment of which therefore would lead to immune dysregulation of all types, including Th2 (56, 69–73).

On this point, it is important to note that Treg failure is always a consideration when trying to understand how impaired TCR signaling could lead to Th2 phenotypes, since an ideal TCR signal is necessary for normal Treg development, differentiation, and function (74). While CARD11DN patient Tregs appeared quantitatively and qualitatively normal, the mouse model suggested otherwise (26). It is further noteworthy that while the mechanism of weak TCR signal failing to curtail STAT5b activity has not yet been studied in the human TCR signaling defects, gain-of-function missense mutations in STAT5b, and JAK1—which activates STAT5b—are associated with syndromes characterized by profound early onset dermatitis and eosinophilia (75, 76). That said, while STAT5bGOF mutations lead to a Th2 phenotype, so too can STAT5bLOF mutations, which are associated with severe Treg impairment (77). While in humans it is difficult to tease apart the relative contributions of effector T cell intrinsic predisposition toward Th2 responses and responsiveness to extrinsic regulation from the number and function of Tregs themselves, it is still important to study both in the context of human diseases of impaired TCR signaling.

## Conclusion

A great deal remains unknown or unproven with respect to the direct role for TCR signaling defects and/or weak TCR signaling in human allergic disease. The limitations which exist when studying human T helper differentiation make it harded to directly demonstrate causality. However, the preponderance of evidence coupling mouse and human *in vitro* studies with *ex vivo* human studies suggests disruption of a number of TCR signaling pathways could well lead to a Th2 phenotype which in turn drives an organismal atopic disease. Apart from the mechanistic insight this provides, how such knowledge could be translated into positive therapeutic manipulation remains a question. Balancing the therapeutic manipulation with risk and cost is of course key. While indeed targeting Th2 cytokines has been quite successful in the clinic, the use of such medications is still in its early phases, and they are extremely expensive. Of course, depending on the severity of disease, bone marrow transplant can be an option, and in theory so could gene therapy and/or gene editing. Other interventions meant to strengthen TCR signaling always run the risk of leaning toward aberrant autoreactivity as well. The ultimate consequences of these balances and their perturbation will be gleaned from continued mechanistic research into the precise mechanisms by which the Th2 phenotypic program emerges when TCR signaling is impaired.

## Author Contributions

The author confirms being the sole contributor of this work and approved it for publication.

## Conflict of Interest Statement

The author declares that the research was conducted in the absence of any commercial or financial relationships that could be construed as a potential conflict of interest. The handling Editor declared a shared affiliation, though no other collaboration, with the author.
